# COVID-19 burden differed by city districts and ethnicities during the pre-vaccination era in Amsterdam, the Netherlands

**DOI:** 10.3389/fpubh.2023.1166193

**Published:** 2023-06-23

**Authors:** Yara Bachour, Elke Wynberg, Liza Coyer, Marcel Buster, Anja Schreijer, Yvonne T. H. P. van Duijnhoven, Alje P. van Dam, Maria Prins, Tjalling Leenstra

**Affiliations:** ^1^Department of Infectious Diseases, Public Health Service of Amsterdam, Amsterdam, Netherlands; ^2^Department of Infectious Diseases, Amsterdam Institute for Infection and Immunity (AII), Amsterdam UMC, University of Amsterdam, Amsterdam, Netherlands; ^3^Department of Epidemiology, Health Promotion and Care Innovation, Public Health Service of Amsterdam, Amsterdam, Netherlands

**Keywords:** SARS-CoV-2, COVID-19, infection, hospitalization, ethnicity, living area, city district

## Abstract

**Background:**

During the first wave of COVID-19 in Amsterdam, the Netherlands, a disproportional number of COVID-19 hospitalizations occurred in individuals with an ethnic minority background and in individuals living in city districts with a lower socioeconomic status (SES). In this study, we assessed whether these disparities continued throughout the second wave, when SARS-CoV-2 testing was available to anyone with symptoms but prior to the availability of COVID-19 vaccination.

**Methods:**

Surveillance data on all notified SARS-CoV-2 cases in Amsterdam between 15 June 2020 and 20 January 2021 were matched to municipal registration data to obtain the migration background of cases. Crude and directly age- and sex-standardized rates (DSR) of confirmed cases, hospitalizations, and deaths per 100,000 population were calculated overall, and by city districts, and migration backgrounds. Rate differences (RD) and rate ratios (RR) were calculated to compare DSR between city districts and migration backgrounds. We used multivariable Poisson regression to assess the association of city districts, migration backgrounds, age, and sex with rates of hospitalization.

**Results:**

A total of 53,584 SARS-CoV-2 cases (median age 35 years [IQR = 25–74]) were notified, of whom 1,113 (2.1%) were hospitalized and 297 (0.6%) deceased. DSR of notified infections, hospitalization, and deaths per 100,000 population were higher in lower SES peripheral city districts (South-East/North/New-West) than higher SES central districts (Central/West/South/East), with almost a 2-fold higher hospitalization DSR in peripheral compared to central districts (RR = 1.86, 95%CI = 1.74–1.97). Individuals with a non-European migration background also had a higher COVID-19 burden, particularly with respect to hospitalization rates, with a 4.5-fold higher DSR for individuals with a non-European background compared to ethnic-Dutch (RR 4.51, 95%CI = 4.37–4.65). City districts, migration backgrounds, male gender, and older age were independently associated with COVID-19 hospitalization rates.

**Discussion:**

Individuals with a non-European background and individuals living in city districts with lower SES continued to independently have the highest COVID-19 burden in the second wave of COVID-19 in Amsterdam, the Netherlands.

## Background

The coronavirus disease 2019 (COVID-19) pandemic, caused by the severe acute respiratory syndrome coronavirus 2 (SARS-CoV-2), emerged in December 2019 in Wuhan, China, and the virus spread globally within months (1, 2). Consequently, the World Health Organization (WHO) declared COVID-19 as a public health emergency of international concern (PHEIC) on 11 March 2020 (3). By 1 January 2023, almost 652 million infections and more than 6.7 million deaths have been reported worldwide (4). The COVID-19 pandemic was declared no longer to be a PHEIC on 5 May 2023 (5).

In the Netherlands, SARS-CoV-2 was first notified in February 2020, after which the first wave of infections occurred until June 2020 (3). In order to mitigate the spread of the virus and relieve pressure on healthcare services, a series of non-pharmaceutical interventions were introduced as part of a national lockdown, including working from home and the closure of many public facilities. By 1 June 2020, testing with polymerase chain reaction (PCR) for SARS-CoV-2 infection was made available to all persons with COVID-19 symptoms, having previously been restricted to a few select groups. The second wave of infections emerged in the Netherlands by the end of August 2020 and persisted beyond January 2021 (6). Again, this led to a set of restrictions, although less stringent than for the first wave (7). From 1 December 2020, close contacts of COVID-19 cases were also eligible for testing on day 5 of quarantine, regardless of their symptoms. The Netherlands implemented COVID-19 vaccination in early January 2021 for healthcare workers and the older adult population. Vaccination was subsequently widely available for decreasing age groups from March 2021 onward (8).

In Amsterdam, the largest city in the Netherlands, more than half of the 900,000 inhabitants have a migration background (9, 10). We previously reported that a disproportional number of persons with a minority ethnic background were hospitalized as a result of SARS-CoV-2 infection during the first wave of COVID-19 in Amsterdam (9). Differences in the age- and sex-standardized COVID-19 hospitalization rates per 100,000 population between city districts and migration background were stark (9): Individuals living in peripheral city districts with a lower socioeconomic status (SES) were hospitalized for COVID-19 almost twice as often as age- and sex-matched individuals living in central, higher SES districts (11). With the assumption that the increased risk of COVID-19 hospitalization was at least partly due to increased exposure as well as an inability to access testing services, targeted programs were rolled out to increase access to information and testing.

It was unclear whether the disparities identified in our initial study persisted during the second wave, prior to the rollout of the national vaccination program. As such, this study compared the rate of notified SARS-CoV-2 infections, and related hospitalization and deaths, between city districts and ethnic backgrounds in Amsterdam between 15 June 2020 and 20 January 2021.

## Methods

### Study design and population

We conducted a registry-based observational study using routinely collected notification data. Eligible for inclusion in this study were all PCR or antigen-confirmed SARS-CoV-2 infections notified to the Public Health Service (PHS) of the Amsterdam-Amstelland region, the Netherlands, between 15 June 2020 and 20 January 2021. We included only individuals residing in the municipality of Amsterdam in this study. No other inclusion criteria were applied.

### Data collection

We extracted COVID-19 surveillance data from the Amsterdam-Amstelland COVID-19 notification database on 21 January 2021. These data included the following: age at the notification, date of birth, sex, postal code, date of symptom onset, notification date, hospitalization, and mortality. Hospitalization and mortality status were recorded using two different sources: (1) the notified case or their contacts during routine contact tracing, or (2) healthcare providers, usually when hospitalization or death occurred days or weeks after initial notification. Case records were therefore updated in the notification database if healthcare providers communicated that the patient had been hospitalized or dead as a result of COVID-19 at a later stage. As a result, all individuals with a notified infection were assumed by the PHS not to be hospitalized and not to have died as a result of COVID-19 unless explicitly informed otherwise. We matched surveillance data to registration data from the municipality database of the City of Amsterdam (BRP) to retrieve the country of birth of each notified patient and their parents.

### Outcomes

We evaluated the number of notified SARS-CoV-2 infections and related hospitalizations and deaths, overall and over time (by notification date as well as the date of symptom onset). Outcomes were as follows: notified SARS-CoV-2 infection (based on a validated positive test result, either PCR or antigen test performed in an authorized testing facility), COVID-19-related hospital admission, and death. Henceforth, we refer to individuals with a notified SARS-CoV-2 infection as confirmed COVID-19 cases.

### Definitions

The city district was determined based on the postal code of the current residence (12) and categorized into Center, New-West, North, East, West, South, and South-East. Based on recent socioeconomic data, residents of the Central, West, South, and East districts have higher average incomes, while the South-East, North, and New-West districts have lower average incomes (13). Migration background and generation were based on the country of birth of individuals and their parents according to national definitions (14). First, we distinguished individuals with and without migration backgrounds. Individuals were considered not to have a migration background (i.e., ethnic-Dutch) if both their parents were born in the Netherlands. Individuals were considered to have a migration background if they were born abroad with at least one parent born abroad (first generation) or if they were born in the Netherlands with both parents born abroad (second generation) (15). In the case of a second-generation migrant, for whom both parents were born abroad, the mother's country of birth was leading in determining the migration background. Furthermore, we then subdivided individuals with a migration background into those originating from a European or non-European country, according to the classification of the Dutch Central Bureau of Statistics (CBS) (16). Exceptions to this rule included migrants originating from North America, Australia, Indonesia, or Japan, who were classified under European due to their country's high socioeconomic status.

Although the second wave started from August 2020 onward, we defined the second wave of infections as the period from June 2020 to January 2021 to perform a consecutive study on our former data.

### Statistical analysis

We calculated crude and directly standardized rates (DSR) of confirmed COVID-19 cases, hospitalizations, and deaths per 100,000 population, overall and by city district and migration background. For the group of non-European migration background, we additionally calculated these outcomes separately for the largest non-European groups in the Netherlands (i.e., Netherlands Antilles, Morocco, Surinam, Turkey, and Ghana). Rates were standardized for age ( ≤ 14, 15–29, 29–44, 45–59, 60–74, and ≥75 years) and sex (female and male) (17). We used the gamma method to calculate 95% confidence intervals (CI) (18, 19). To evaluate both relative and absolute differences between groups, we computed rate differences (RD) and rate ratios (RR) to compare the DSR of hospitalization between (i) city districts, (ii) migration backgrounds (first- and second-generation migrants combined), and (iii) a combination of both variables, i.e., six strata of city districts (dichotomized into the central districts with higher average incomes [Central/West/South/East] and peripheral districts with lower average incomes [Southeast/North/New-West]) and migration background (none [ethnic-Dutch], European, and non-European) (13). We combined first and second generations as this was highly correlated with age and a stratified analysis would result in low numbers in specific age strata. Only individuals with available data on both city districts (i.e., postal code of the residence) and migration statuses (i.e., were able to be linked to municipality records) were included in the comparative analyses.

A sensitivity analysis was performed where the DSR for confirmed COVID-19-related hospitalizations were restricted to the population aged < 60 years. The purpose of this analysis was to evaluate the possible confounding effect of receiving formal home care or living in a nursing home on the association between migration background and COVID-19 hospitalization risk.

A previous study indicates that migration background is correlated with residing in peripheral city districts with lower socioeconomic status than central city districts (20). We, therefore, wished to evaluate the independent effects of city districts (peripheral and central) and migration backgrounds (none [ethnic-Dutch], European, and non-European). We did so using a Poisson regression model, adjusting for age and sex, and using the log of the population size per district/migration background/sex/age stratum as an offset. In this model, we additionally evaluated the possible effect modification of the city district on migration backgrounds by adding an interaction term between them, which was tested for statistical significance using a likelihood ratio test (LRT).

In the epidemiological curve by date of the onset of symptom, missing dates were imputed, assuming that all those who were tested had symptoms. First, we created a distribution of time between symptom onset and case notification for those with a known date of symptom onset. Second, we randomly sampled from this distribution to estimate the date of the onset of the symptom if this was missing.

We assumed statistical significance at a *P*-value < 0.05. We used the dsr package in R to calculate DSR, RD, and RR (19). All analyses were performed in R (version 3.6.3, Vienna, Austria).

## Results

### Study population

**Figure 1:**
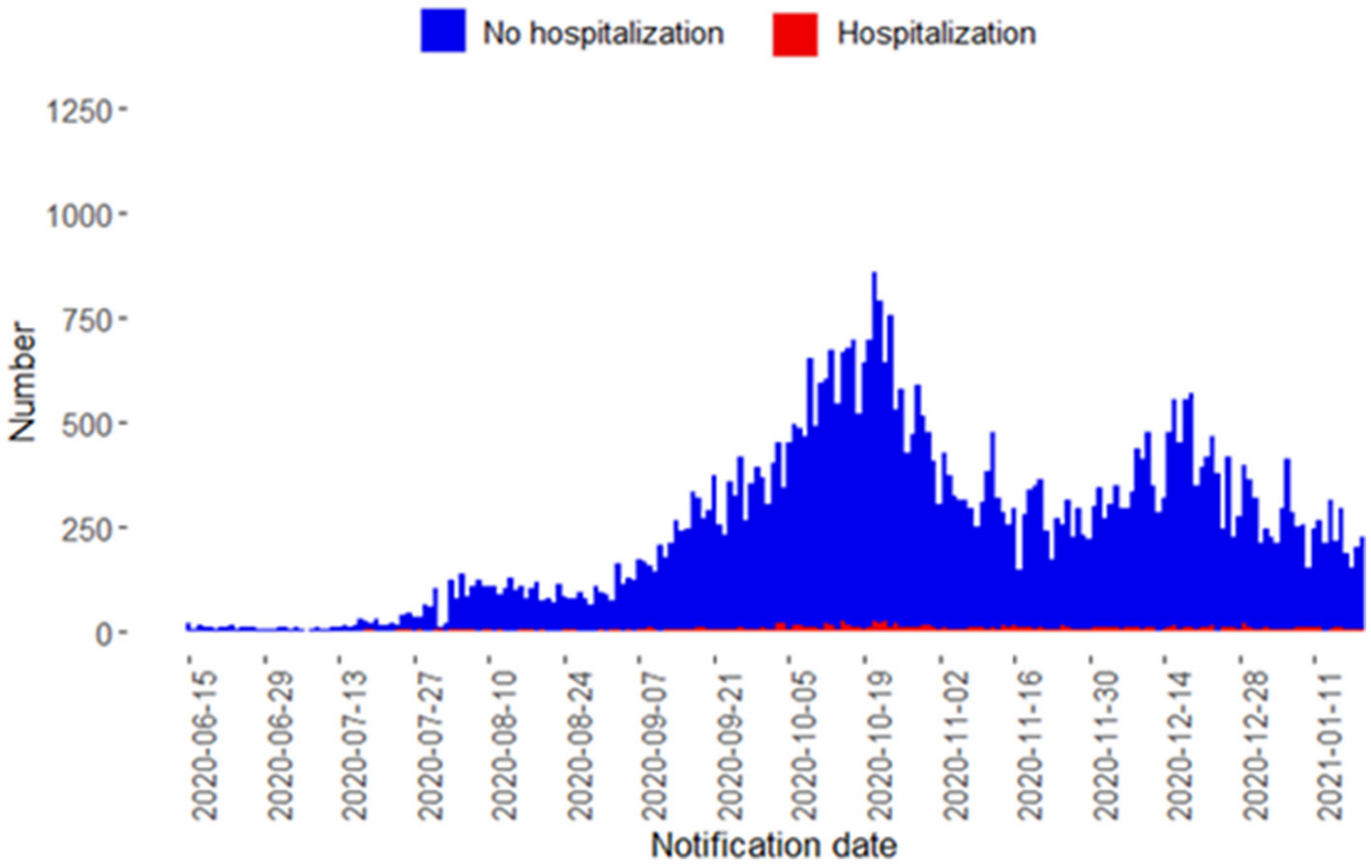
Confirmed COVID-19 cases notified to the Public Health Service of Amsterdam by notification date from June 15, 2020, to January 20, 2021, stratified by hospitalization status.

Between 15 June 2020 and 20 January 2021, a total of 53,584 COVID-19 cases were notified. Among these individuals, 1,113 (2.1%) were hospitalized and 297 (0.6%) deceased. Figure 1 shows that there were two distinct peaks of notified COVID-19 cases during the second COVID-19 wave: in the middle of October and December. Characteristics of COVID-19 cases by hospitalization status are shown in Table 1. Hospitalized cases were older than non-hospitalized cases (median 65 years [IQR = 50–76] vs. 34 years [IQR = 25–51]). Confirmed cases were slightly more often female than male (28,299 [52.8%]), while hospitalized cases were more often male (636/1,113 [57.1%]).

**Table 1:** Characteristics of confirmed COVID-19 cases in Amsterdam, the Netherlands, 15 June 2020–20 January 2021, by COVID-19 hospitalization status.

	**Confirmed COVID-19 cases (*****N** =* **53,584)**		**Hospitalized (*****N** =* **1,113)**		**Not hospitalized (*****N** =* **52,471)**	
**Characteristic**	**N**	**%**	**n**	**%**	**n**	**%**
**Age in years, median [IQR]**	35	[25–52]	65	[50–76]	34	[25–51]
**Sex**						
Female	28,299	52.8	477	42.9	27,822	53.0
Male	25,256	47.1	636	57.1	24,620	46.9
Unknown	29	0.1	0	0	29	0.1
**City district**						
Center	4.363	8.1	46	4.1	4,317	8.2
New-West	11.859	22.1	318	28.6	11,541	22.0
North	6,148	11.5	125	11.2	6,023	11.5
East	8,246	15.4	151	13.6	8,095	15.4
West	8,819	16.4	176	15.8	8,643	16.5
South	7,817	14.6	112	10.1	7,705	14.7
South-East	6,253	11.7	184	16.5	6,069	11.6
Other/unknown	79	0.1	1	0.1	78	0.1
**Died**						
Yes	297	0.6	115	10.3	182	0.4
No/unknown	53,287	99.5	998	89.7	52,289	99.7

IQR, interquartile range.

**Figure 2:**
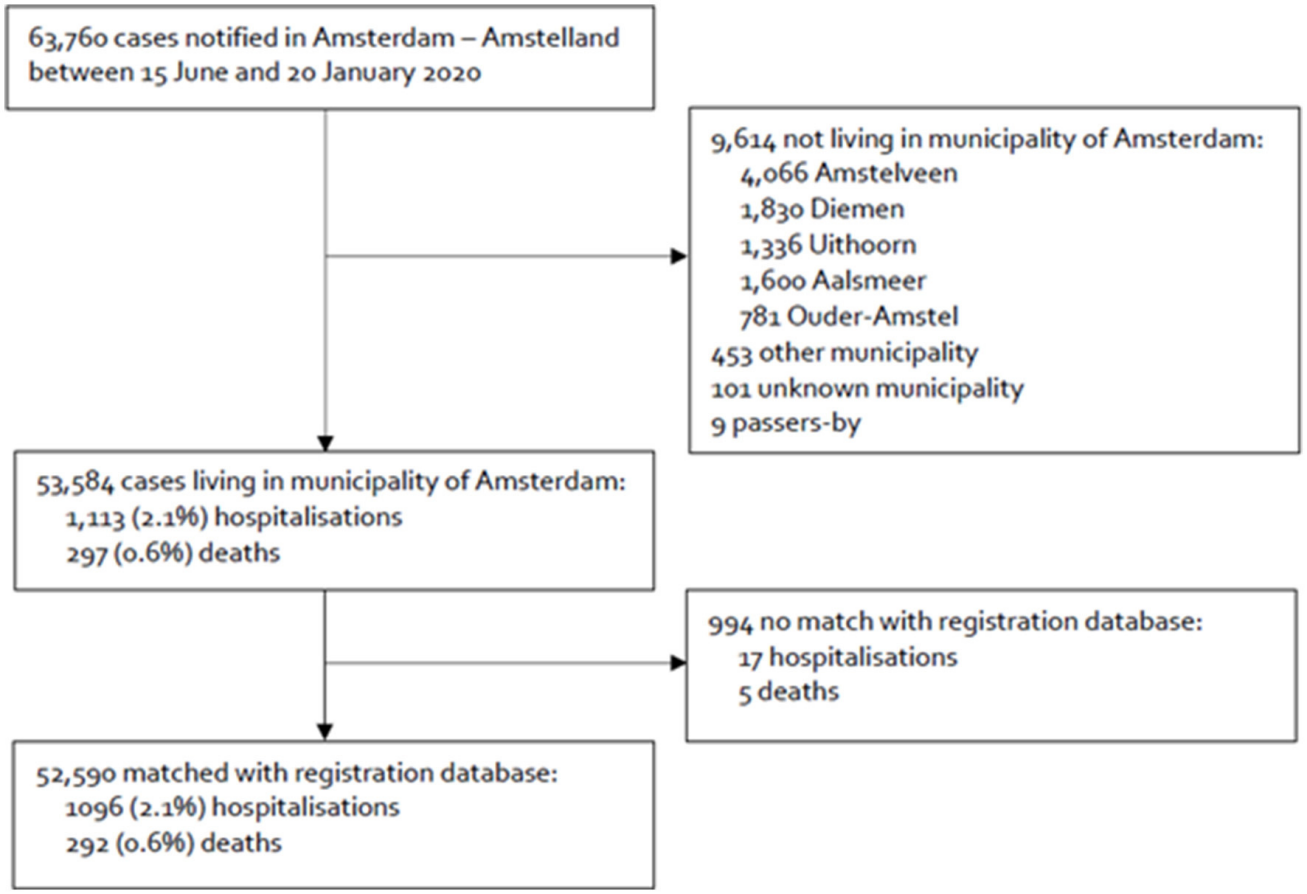
Flowchart of the inclusion of confirmed COVID-19 cases notified to the Public Health Service of Amsterdam, the Netherlands, between 15 June 2020 and 20 January 2021, and linkage of notification data with the municipality register.

In total, 52,590 of 53,584 confirmed COVID-19 cases (98.1%) could be matched to the Amsterdam municipality registration database, including 1,096 of 1,113 hospitalizations (98.5%) and 292 of 297 deaths (98.3%) (Figure 2).

### Rates of confirmed COVID-19 case notifications

Supplementary Tables S1, S2 show the DSR of confirmed COVID-19 cases per 100,000 population by city district and migration background. After standardizing for age and sex, the rate of confirmed COVID-19 cases per 100,000 population was significantly higher in peripheral city districts compared to central districts (RR = 1.30, 95%CI = 1.28–1.31). DSR of confirmed cases was the highest in individuals living in New-West (RR = 1.56, 95%CI = 1.53–1.60) and South-East (RR = 1.45, 95%CI = 1.41–1.49), compared to the residents of the city center (Supplementary Table S1). Individuals with a non-European background had a 1.4-fold higher DSR of notified infection compared to individuals without a migration background (95%CI = 1.40, 95%CI = 1.38–1.41), while individuals with a European migration background had a lower DSR compared to individuals without a migration background (RR = 0.72, 95%CI = 0.69–0.74). DSR of notified infections was highest in individuals of Moroccan (RR = 1.88, 95%CI = 1.86–1.91) and Turkish origin (RR = 1.86, 95%CI = 1.82–1.89) (Supplementary Table S2).

### Hospitalization rates

Table 2 shows hospitalization DSR per 100,000 population by city district and migration background. Hospitalization DSR were almost two-fold higher among individuals living in peripheral city districts compared to central districts (RR = 1.86, 95%CI = 1.74–1.97), with 80 additional hospitalizations per 100,000 population (RD = 80, 95%CI = 64–96). Supplementary Table S3 provides further details per individual city district. We observed a 4.5-fold higher hospitalization DSR in individuals with a non-European migration background compared to ethnic-Dutch individuals (RR 4.51, 95%CI = 4.37–4.65), with 213 additional hospitalizations per 100,000 population (RD 213, 95%CI = 190–235 per 100,000 population). Individuals with a European migration background also had a higher DSR compared to individuals without a migration background (RR 1.36 95%CI = 1.14–1.58). When comparing specific non-European migrant groups to the ethnic-Dutch population, the highest hospitalization RR were among individuals of Moroccan (RR 6.64, 95%CI = 6.46–6.81; RD = 341, 95%CI = 287–395 per 100,000 population) and Turkish (RR 5.93, 95%CI = 5.7–6.16; RD = 299, 95%CI = 228–369 per 100,000 population) migration backgrounds (Supplementary Table S4).

**Table 2:** Hospitalization rates by city district and migration background among confirmed COVID-19 cases who were linked to the population register in Amsterdam, the Netherlands, from 15 June 2020 to 20 January 2021.

	**Hospital admissions**	**Population^a^**	**Crude rate per 100,000 population (95% CI)**	**Standardized rate per 100,000 population^b^ (95% CI)**	**Standardized rate difference (95% CI)**	**Standardized rate ratio (95% CI)**
**Total**	1,096	873,055	125.54 (118.21–133.19)			
**City district** ^c^						
Central	480	523,536	91.68 (83.66–100.27)	93.41 (85.24–102.17)	Ref.	Ref.
Peripheral	615	349,519	175.96 (162.32–190.43)	173.31 (159.85–187.6)	79.89 (63.81–95.98)	1.86 (1.74–1.97)
**Migration background**						
None (ethnic-Dutch)	292	386,521	75.55 (67.13–84.73)	60.53 (53.65–68.04)	Ref.	Ref.
Non-European	681	316,720	215.02 (199.17–231.79)	273.22 (252.21–295.51)	212.69 (190.13–235.26)	4.51 (4.37–4.65)
European	116	169,814	68.31 (56.45–81.93)	82.36 (67.86–99.03)	21.83 (5.06–38.6)	1.36 (1.14–1.58)

a Population on April 1, 2020.

b Standardized for age (in 15-year groups) and gender, using the total population of Amsterdam as the standard population.

c One matched case had missing data.

**Figure 3:**
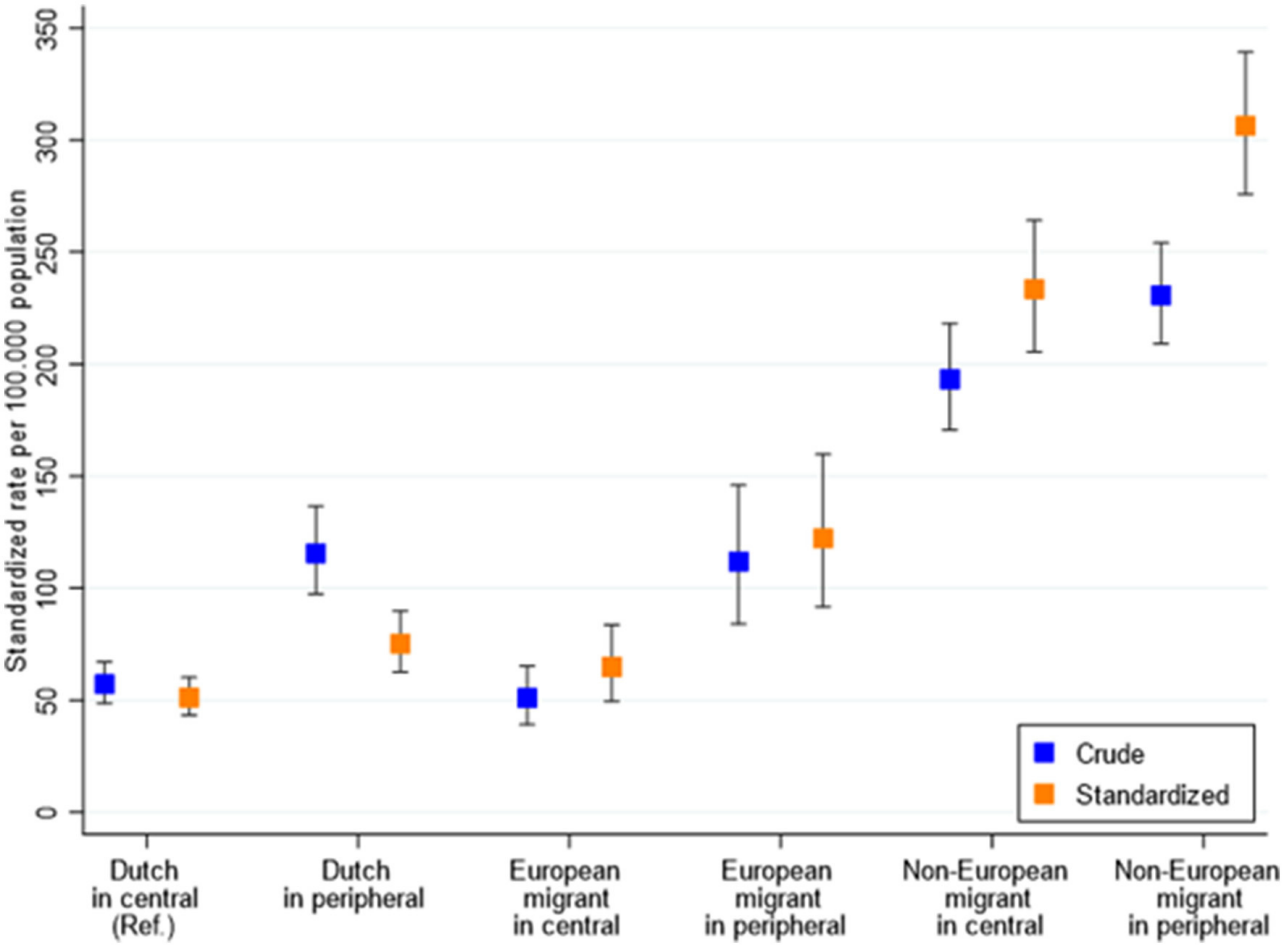
Age- and sex-standardized hospitalization rates per 100,000 population in Amsterdam, the Netherlands, between 15 June 2020 and 20 January 2021, stratified by migration background and city district of residence. Central city districts (higher SES) = Central, West, South, and East districts with higher average incomes; peripheral city districts (lower SES) = South-East, North, and New-West. European migration background included migrants originating from North America, Australasia, Indonesia, or Japan, due to their high socioeconomic status compared to non-European countries.

**Figure 4:**
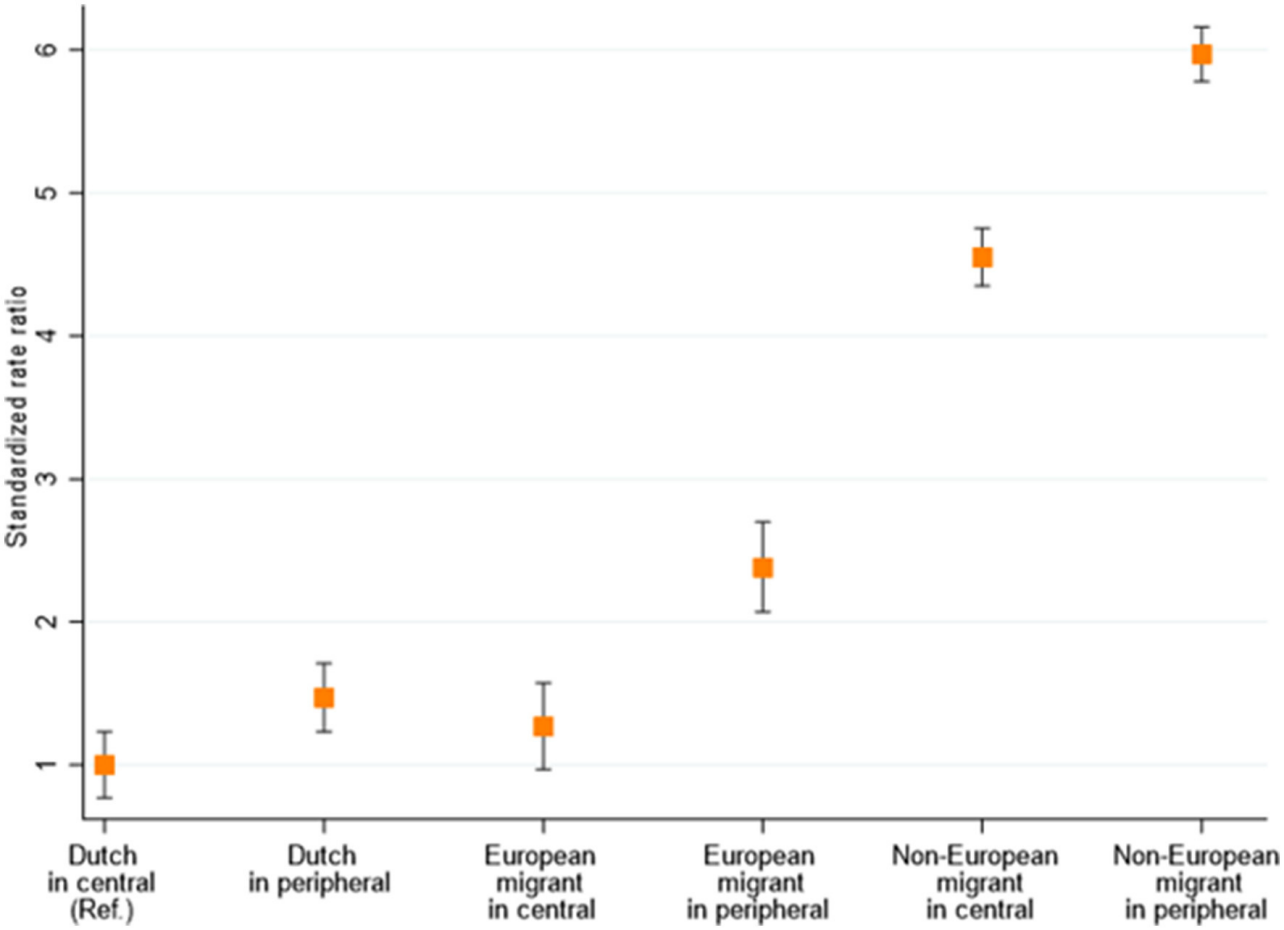
Age- and sex-standardized rate ratios of COVID-19 hospitalization in Amsterdam, the Netherlands, between 15 June 2020 and 20 January 2021, stratified by migration background and city district of residence. Central city districts (higher SES) = Central, West, South, and East districts with higher average incomes; peripheral city districts (lower SES) = South-East, North, and New-West. European migration background included migrants originating from North America, Australasia, Indonesia, or Japan, due to their high socioeconomic status compared to non-European countries.

When comparing differences in hospitalization DSR by both city district and migration background, the DSR was the highest in non-European residents of peripheral districts (DSR 306 hospitalizations per 100,000 population [95%CI 276–339]), who had a six times higher hospitalization DSR than ethnic-Dutch residents of central districts (RR 5.97 [95%CI 5.78–6.16]) (Figures 3, 4).

### Death rates

Supplementary Tables S5, S6 show death DSR per 100,000 population by city district and migration background in the population matched to the population registry. Mortality DSR was 1.4 times higher among individuals living in peripheral compared to central city districts (RR 1.40, 95%CI = 1.18–1.64). Specifically, mortality DSR was the highest in individuals living in New-West (RR = 2.32, 95%CI = 1.80–2.84) and West (RR = 2.26, 95%CI = 1.71–2.80) compared to the city center. When comparing death DSR in individuals with a non-European background to individuals without a migration background, this reveals a 2-fold higher DSR in the non-European group (RR 1.98, 95%CI = 1.73–2.23). Similar to DSR for infections and hospitalization, individuals of Moroccan heritage (RR = 2.61, 95%CI = 2.23–3.00) and Turkish origin (RR = 2.44, 95%CI = 1.92–2.97) had the highest death rates compared to the ethnic-Dutch population, among the largest non-European migrant populations.

### Sensitivity analysis

When restricting to confirmed COVID-19 cases under 60 years, individuals with a non-European migration background continued to have an almost five times greater risk of hospitalization, compared to ethnic-Dutch (RR 4.86, 95%CI = 4.59–5.12) (Supplementary Table S7).

### Regression analyses

Multivariable Poisson regression revealed that having a migration background, living in a peripheral city district, male sex, and older age were independently associated with higher DSR hospitalization rates (Table 3). No statistically significant interaction was found between migration background and city district (*p* = 0.21).

**Table 3:** Multivariable Poisson regression model of factors associated with hospitalization among confirmed COVID-19 cases linked to the population register in Amsterdam, the Netherlands, 15 June 2020–20 January 2021.

**Characteristic**	**aRR (95% CI)**	***P*-value**
**Migration background**		< 0.01
None (ethnic-Dutch)	1 (Ref.)	
European	1.41 (1.13–1.75)	
Non-European	4.27 (3.71–4.93)	
**City district**		< 0.01
Central (C/W/S/E)	1 (Ref.)	
Peripheral (SE/N/NW)	1.42 (1.26–1.41)	
**Sex**		< 0.01
Male	1 (Ref.)	
Female	0.70 (0.62–0.79)	
**Age, years**		< 0.01
< 45	1 (Ref.)	
45–59	3.25 (2.68–3.94)	
60–74	9.81 (8.29–11.63)	
≥75	26.28 (22.02–31.43)	

aRR, adjusted rate ratio; CI, confidence interval. Estimates were obtained from a multivariable Poisson regression model. We tested for interaction between migration background and city district, but this was not significant (*p* = 0.12) so was excluded from the final model. Incidence rates in the Poisson regression model were calculated with the Amsterdam population as the denominator.

## Discussion

This study reveals ethnic and geographical differences during the COVID-19 pandemic in Amsterdam during the second wave of COVID-19, prior to the rollout of vaccination. Age- and sex-standardized rates of confirmed COVID-19 cases, hospitalizations, and deaths were higher in lower SES peripheral city districts than in higher SES central districts. Additionally, the COVID-19 burden was significantly higher among individuals with a non-European migration background compared to Dutch ethnic origin. The current results corroborate our earlier findings of higher rates of hospitalizations for people living in peripheral city districts and with a non-European migration background (8). However, while the differences in hospitalization between city districts seemed constant, differences between migration backgrounds amplified during the second wave. While individuals with a non-European migration background were twice as likely to be hospitalized compared to individuals without a migration background during the first wave (8), this increased to 4.5 times during the second wave. Specifically, we observed the greatest increases in relative risk for individuals of Moroccan and Turkish ethnic origin.

Differences in hospitalization rates between the first and second waves could be explained in multiple ways. First, Dutch non-pharmaceutical interventions during the first wave were more restrictive than during the second wave. We speculate that fewer general restrictions and increased exposure may consequently have augmented differences in exposure risk between populations with a non-European migration background compared to ethnic-Dutch individuals, accentuating existing differences in COVID-19 hospitalization rates between ethnic groups (21). In addition, early intervention in the community to prevent COVID-19 hospital admission was inaccessible to all during the first wave of COVID-19 but became more prevalent during the second wave due to increased expertise among general practitioners on the clinical management of COVID-19 in the community (22). This may have served to prevent more hospitalizations among ethnic-Dutch confirmed COVID-19 cases than those with a non-European migration background, thus strengthening the differences in hospitalization risk between these groups as the pandemic progressed.

Differences in SARS-CoV-2 infections and related hospitalization and deaths, among people with a migration background and lower SES, have been observed in the Netherlands as well as globally (23). Similar to our results, Chilunga et al. observed a higher death rate among patients with a migration background compared to patients of Dutch origin, mainly among those with Turkish, Moroccan, and Surinamese backgrounds (24). This was almost 30% higher in the second wave than in the first wave. The authors postulate that the latter might be due to elevated SARS-CoV-2 infections among migration groups. Globally, similar results were seen. A recent meta-analysis showed that ethnic inequalities in COVID-19 health outcomes are seen. This review revealed an increased risk for COVID-19 infections among people from Black (adjusted risk ratio [aRR]:1.78, 95% CI:1.59–1.99), South Asian (aRR:3.00, 95% CI:1.59–5.66), Mixed (aRR:1.64, 95% CI:1.02–1.67), and Other ethnic groups (aRR:1.36, 95% CI:1.01–1.82) compared with White majority populations. Almost all minority ethnic groups were at increased risk of hospital admission and ICU admission, and higher death rates were mostly seen among patients with Hispanic, Mixed, and Indigenous groups. The authors suggest that these differences might be due to several factors, such as socioeconomic inequalities, cultural factors, and barriers to adequate healthcare.

Nonetheless, it is important to discuss the multitude of complex factors that may contribute to increased COVID-19 burden in peripheral city districts as well as among key non-European ethnic groups, to help design tailored interventions. Indeed, while belonging to an ethnic minority group is associated with having a lower socioeconomic status, migration background has consistently been shown to be an additional, independent determinant of COVID-19 hospitalization (25)—also shown in the current analysis. Socioeconomic deprivation is likely to facilitate viral spread due to working in professions with a higher likelihood of exposure to infection, more frequently using public transportation, and living in small houses with larger families (26–28). It has also been well-documented that lower socioeconomic status is strongly correlated with an increased burden of non-communicable disease, which in turn amplifies severe outcomes during SARS-CoV-2 infection (29, 30). The added, increased risk of migration background for COVID-19 hospitalization is likely attributable to various factors that could influence both exposure to SARS-CoV-2 and subsequent severe disease. For instance, living in intergenerational familial units or frequent gatherings of extended families or religious communities may lead to more rapid viral spread (31, 32). Barriers to early intervention by community healthcare services due to language or cultural differences (33, 34) can increase the risk of hospitalization, as well as a higher prevalence of comorbidities.

Cultural differences might also underpin the likelihood for ethnic-Dutch older adults to reside in nursing homes where supportive care can be provided for COVID-19 complications, compared to older adults in non-European communities who may be more often cared for at home, with an increased chance for hospital admission when experiencing severe COVID-19 symptoms (35). Interestingly, a recent study in the Netherlands found that while migration background was associated with an increased risk of COVID-19 hospitalization, 21-day mortality and ICU admission rates were comparable between different ethnic groups once hospitalized (28). These findings suggest that relative rates of hospitalization and mortality should be similar between ethnic groups. In our study, however, we observed greater differences in hospitalization rates compared to mortality rates. This adds further evidence to suggest that COVID-19 mortality in the community may be underreported in those with a non-European migration background dying at home without having been tested, as opposed to ethnic-Dutch older adults residing in nursing homes. When restricting to individuals aged younger than 60 years, however, we found the relative risk for COVID-19 hospitalization among non-European individuals to be no different than in the primary analysis (RR 4.86, 95%CI = 4.59–5.12 <60 years; compared to RR 4.51, 95%CI = 4.37–4.65 overall). The interplay between predisposing social, economic, cultural, and medical factors for COVID-19 is therefore complex. To alleviate the COVID-19 burden, targeted information and testing campaigns as well as long-term, parallel investments to reduce both socioeconomic deprivation and non-communicable disease prevalence are crucial.

Following the first wave of COVID-19, several targeted prevention programs were undertaken by the Public Health Service of Amsterdam (36). First, in partnership with existing research projects, focus groups were conducted in October 2020 with numerous key non-European migrant groups to evaluate the key barriers to adhering to COVID-19 regulations, thus increasing exposure to and onward transmission of the virus. Second, a corona prevention team was set up to improve the inclusivity of COVID-19 public health communication tools and initiate specific interventions, such as mobile testing services, in high-incidence city districts (37) given that access to testing services has been previously reported to be lower among ethnic minority groups (38, 39). It is remarkable that despite these programs, differences in the risk of hospitalization between groups appear to have increased in the current analysis compared to our previous analysis. This suggests that these programs might not have been completely effective in preventing further spread and infection among ethnic groups, although we cannot be sure whether the differences observed may have been even greater without these interventions. Further efforts are therefore needed to understand the drivers of and identify effective interventions to combat the disproportionate burden of COVID-19 in Amsterdam. This will allow policymakers to invest in ways to prevent these inequalities in a future outbreak or pandemic.

Our study's strengths are the use of notification data in the period prior to the availability of vaccinations and self-tests, thus allowing for the analysis of the COVID-19 burden without biases from pharmaceutical interventions. Moreover, the vast majority of COVID-19 cases were linked to the municipality register, resulting in few missing data on city districts and migration backgrounds. However, our study also has several limitations. First, as we used confirmed COVID-19 notifications, a detection bias exists, driven by access to testing facilities. Second, imputing the date of illness onset based on the distribution of time from illness onset to notification date for individuals missing this information may have led to a small proportion of asymptomatic contacts being erroneously allocated an earlier date of illness onset. However, given that this misclassification is unlikely to be differential and only applicable to a small proportion of individuals, no impact on our conclusions is expected. Third, hospitalization status might not have been accurately recorded for all individuals, for instance, if contact-tracing teams were unable to contact the patient or their relatives and hospital reporting of admission was incomplete. Furthermore, some hospitalizations may have been due to reasons other than COVID-19. However, given that under- or over-reporting of hospitalization status is unlikely to be linked to city district or migrations status, any influence on the measures of the effect of these factors is likely to have been minimal. Additionally, we did not have access to individual-level data on possible causal or confounding factors such as comorbidities and occupation. Further studies should focus on studying individual and community-level drivers of COVID-19 disparities.

## Conclusion

Despite the initiation of public health programs to reduce disparities in the COVID-19 burden, the risk of SARS-CoV-2 infection, hospitalization, and deaths continued to be higher in peripheral city districts with a lower SES as well as in individuals with a non-European migration background during the second wave. Socioeconomic, cultural, and medical factors may account for a large part of these differences. Prospective observational studies are required to evaluate existing strategies and successfully identify and serve groups at increased risk of SARS-CoV-2 infection, severe outcomes, and post-acute COVID-19 syndrome as we move forward from the pandemic.

## Data availability statement

The original contributions presented in the study are included in the article/Supplementary material, further inquiries can be directed to the corresponding author.

## Ethics statement

Ethical review and approval was not required for the study on human participants in accordance with the local legislation and institutional requirements. Written informed consent for participation was not required for this study in accordance with the national legislation and the institutional requirements.

## Author contributions

TL, MP, LC, and MB conceptualized the study. LC conducted data cleaning and data analysis. TL supervised data analysis. YB, LC, EW, MB, MP, YD, and TL contributed to the interpretation of the results. YB wrote the main manuscript text under the supervision of EW and TL. YB, LC, EW, MB, MP, AS, YD, AD, and TL read the manuscript, provided feedback, and approved the final version. All authors contributed to the article and approved the submitted version.
