# Supplementary material for: COVID-19 burden differed by city districts and ethnicities during the pre-vaccination era in Amsterdam, the Netherlands
Source: Front Public Health. 2023 Jun 23;11:1166193. doi: 10.3389/fpubh.2023.1166193 (PMC10326320; doi:10.3389/fpubh.2023.1166193)
Supplement: Supplementary file 1 [file Table_1.DOCX]

**Supplement to: COVID-19 burden differed by city district and ethnicity during the pre-vaccination era in Amsterdam, the Netherlands**

Yara Bachour, Elke Wynberg, Liza Coyer, Marcel Buster, Anja Schreijer, Yvonne T.H.P. van Duijnhoven, Alje P. van Dam, Maria Prins and Tjalling Leenstra

**Supplemental Materials**

| **Overview of Contents** | **Page** |
| --- | --- |
| **Supplementary Tables** |  |
| Supplementary Table S1. Infection rates by city district among those linked to the registration database, Amsterdam, The Netherlands, 15 June 2020-20 January 2021 | 2 |
| Supplementary Table S2. Infection rates by migration history (first and second generation combined) among those linked to the registration database, Amsterdam, The Netherlands, 15 June 2020-20 January 2021 | 3 |
| Supplementary Table S3. Hospitalisation rates by city district among those linked to the registration database, Amsterdam, the Netherlands, 15 June 2020-20 January 2021 | 4 |
| Supplementary Table S4. Hospitalisation rates by migration history (first and second generation combined) among those linked to the registration database, Amsterdam, the Netherlands, 15 June 2020-20 January 2021 | 5 |
| Supplementary Table S5. Death rates by city district among those linked to the registration database, Amsterdam, The Netherlands, 15 June 2020-20 January 2021 | 6 |
| Supplementary Table S6. Death rates by migration history (first and second generation combined) among those linked to the registration database, Amsterdam, The Netherlands, 15 June 2020-20 January 2021 | 7 |
| Supplementary Table S7. Hospitalisation rates under 60 years of age by migration history (first and second generation combined) among those linked to the registration database, Amsterdam, the Netherlands, 15 June 2020-20 January 2021 | 8 |

**Supplementary Table S1. Infection rates by city district among those linked to the registration database, Amsterdam, The Netherlands, 15 June 2020-20 January 2021**

|  | **Infections** | **Population ^a^** | **Crude rate per 100,00 population**  **(95% CI)** | **Standardised rate per 100,00 population ^b^**  **(95% CI)** | **Standardised rate difference (95% CI)** | **Standardised rate ratio**  **(95% CI)** |
| --- | --- | --- | --- | --- | --- | --- |
| **Total** | 52556 | 873,055 | 41875,06 |  |  |  |
|  |  |  |  |  |  |  |
| **City district^c^** |  |  |  |  |  |  |
| Centre | 4255 |  | 4878,3 | 4785,04 | Ref. | Ref. |
|  |  | 87,223 | (4732,81-5027,12) | (4640,14-4933,32) |  |  |
| New-West | 11662 |  | 7283,51 | 7483,38 | 2698,34 | 1,56 |
|  |  | 160,115 | (7151,92-7416,93) | (7347,77-7620,87) | (2498,65-2898,02) | (1,53-1,6) |
| North | 6058 |  | 6075,92 | 6358,61 | 1573,57 | 1,33 |
|  |  | 99,705 | (5923,87-6230,89) | (6197,98-6522,35) | (1355,75-1791,39) | (1,29-1,37) |
| East | 8149 |  | 5738,29 | 5693,92 | 908,87 | 1,19 |
|  |  | 142,011 | (5614,37-5864,25) | (5570,59-5819,29) | (717,32-1100,42) | (1,15-1,23) |
| West | 8662 |  | 5859,39 | 5742,45 | 957,4 | 1,2 |
|  |  | 147,831 | (5736,64-5984,11) | (5620,85-5866,02) | (766,98-1147,83) | (1,16-1,24) |
| South | 7664 |  | 5232,44 | 5223,81 | 438,76 | 1,09 |
|  |  | 146,471 | (5115,94-5350,92) | (5107,12-5342,49) | (251,45-626,08) | (1,05-1,13) |
| South-East | 6106 |  | 6807,21 | 6935,88 | 2150,84 | 1,45 |
|  |  | 89,699 | (6637,53-6980,13) | (6761,47-7113,66) | (1922,54-2379,14) | (1,41-1,49) |

^a^ Population on 1 April 2020

^b^ Standardised for age (in 15-year groups) and gender, using the total population of Amsterdam as the standard population

^c^ 1 matched case had missing data

**Supplementary Table S2. Infection rates by migration history (first and second generation combined) among those linked to the registration database, Amsterdam, The Netherlands, 15 June 2020-20 January 2021**

|  | **Infections** | **Population ^a^** | **Crude rate per 100,00 population**  **(95% CI)** | **Standardised rate per 100,00 population ^b^**  **(95% CI)** | **Standardised rate difference (95% CI)** | **Standardised rate ratio**  **(95% CI)** |
| --- | --- | --- | --- | --- | --- | --- |
| **Total** | 52579 | 873055 | 6022,42 |  |  |  |
|  |  |  |  |  |  |  |
| **Migration background** |  |  |  |  |  |  |
| Netherlands Antilles | 762 | 12126 | 6284.02 | 6259,21 | 650,48 | 1,12 |
|  |  |  | (5845.7-6746.49) | (5818,93-6723,97) | (195,75- 1105,21) | (1,04- 1,19) |
| Morocco | 7523 | 77213 | 9743.18 | 10562,4 | 4953,67 | 1,88 |
|  |  |  | (9524.24-9965.88) | (10319,3-10809,77) | (4697,48- 5209,85) | (1,86- 1,91) |
| Surinam | 4991 | 63944 | 7805.27 | 7607,68 | 1998,95 | 1,36 |
|  |  |  | (7590.21-8024.87) | (7392,86-7827,15) | (1769,48-2228,42) | (1,32- 1,39) |
| Turkey | 4539 | 44417 | 10219.06 | 10414,03 | 4805,3 | 1,86 |
|  |  |  | (9923.91-10520.76) | (10102,56-10732,68) | (4482,29-5128,32) | (1,82- 1,89) |
| Ghana | 602 | 12883 | 4672.82 | 4806,45 | -802,28 | 0,86 |
|  |  |  | (4306.95-5061.47) | (4411,66-5227,09) | (-1212,67-  -391,89) | (0,77- 0,94) |
| Other non-European | 5820 | 106137 | 5483.48 | 5564,44 | -44,29 | 0,99 |
|  |  |  | (5343.5-5626.2) | (5410,3- 5721,86) | (-217,34-  128,75) | (0,96- 1,02) |
| Total non-European | 24286 | 316720 | 7667.97 | 7826,43 | 2217,7 | 1,4 |
|  |  |  | (7571.83-7765.03) | (7726,55-7927,26) | (2091,63-2343,76) | (1,38- 1,41) |
| Total European | 7035 | 169814 | 4142.77 | 4018,04 | -1590,69 | 0,72 |
|  |  |  | (4046.52-4240.73) | (3922,98- 4114,82) | (-1713,15-  -1468,22) | (0,69- 0,74) |
| None (Ethnic-Dutch) | 21076 | 386521 | 5452.74 | 5608,73 | Ref. | Ref. |
|  |  |  |  | (5532,47-5685,78) |  |  |

^a^ Population on 1 April 2020

^b^ Standardised for age (in 15-year groups) and gender, using the total population of Amsterdam as the standard population

**Supplementary Table S3. Hospitalisation rates by city district among those linked to the registration database, Amsterdam, the Netherlands, 15 June 2020-20 January 2021**

|  | **Hospital admissions** | **Population ^a^** | **Crude rate per 100,00 population**  **(95% CI)** | **Standardised rate per 100,00 population ^b^**  **(95% CI)** | **Standardised rate difference (95% CI)** | **Standardised rate ratio**  **(95% CI)** |
| --- | --- | --- | --- | --- | --- | --- |
| **Total** | 1,096 | 873,055 | 125.54 |  |  |  |
|  |  |  | (118.21-133.19) |  |  |  |
| **City district^c^** |  |  |  |  |  |  |
| Centre | 46 |  | 52.74 | 47.68 | Ref. | Ref. |
|  |  | 87,223 | (38.61-70.35) | (34.83-63.73) |  |  |
| New-West | 312 |  | 194.86 | 199.82 | 152.14 | 4.19 |
|  |  | 160,115 | (173.84-217.73) | (178.19-223.36) | (125.91-178.36) | (3.88-4.5) |
| North | 123 |  | 123.36 | 112.17 | 64.48 | 2.35 |
|  |  | 99,705 | (102.53-147.19) | (93.03-134.08) | (40.12-88.85) | (2.01-2.69) |
| East | 148 |  | 104.22 | 114.88 | 67.2 | 2.41 |
|  |  | 142,011 | (88.1-122.43) | (96.96-135.16) | (43.92-90.47) | (2.08-2.74) |
| West | 174 |  | 117.7 | 136.91 | 89.23 | 2.87 |
|  |  | 147,831 | (100.86-136.55) | (117.07-159.15) | (64.37-114.08) | (2.54-3.2) |
| South | 112 |  | 76.47 | 69.52 | 21.84 | 1.46 |
|  |  | 146,471 | (62.96-92.01) | (57.13-83.81) | (2.82-40.85) | (1.11-1.8) |
| South-East | 180 |  | 200.67 | 199.72 | 152.04 | 4.19 |
|  |  | 89,699 | (172.43-232.22) | (171.28-231.53) | (119.41-184.66) | (3.86-4.51) |

^a^ Population on 1 April 2020

^b^ Standardised for age (in 15-year groups) and gender, using the total population of Amsterdam as the standard population

^c^ 1 matched case had missing data

**Supplementary Table S4.** **Hospitalisation rates by migration history (first and second generation combined) among those linked to the registration database, Amsterdam, the Netherlands, 15 June 2020-20 January 2021**

|  | **Hospital admissions** | **Population ^a^** | **Crude rate per 100,00 population**  **(95% CI)** | **Standardised rate per 100,00 population ^b^**  **(95% CI)** | **Standardised rate difference (95% CI)** | **Standardised rate ratio**  **(95% CI)** |
| --- | --- | --- | --- | --- | --- | --- |
| **Total** | 1,096 | 873,055 | 125.54 |  |  |  |
|  |  |  | (118.21-133.19) |  |  |  |
| **Migration background** |  |  |  |  |  |  |
| Netherlands Antilles | 24 | 12,126 | 197.92 | 228.46 | 167.93 | 3.77 |
|  |  |  | (126.81-294.49) | (144.26-343.84) | (73.59-262.27) | (3.35-4.2) |
| Morocco | 235 | 77,213 | 304.35 | 401.64 | 341.12 | 6.64 |
|  |  |  | (266.68-345.86) | (349.65-459.2) | (286.86-395.38) | (6.46-6.81) |
| Surinam | 167 | 63,944 | 261.17 | 237.47 | 176.94 | 3.92 |
|  |  |  | (223.06-303.92) | (202.06-277.3) | (139.43-214.46) | (3.73-4.12) |
| Turkey | 117 | 44,417 | 263.41 | 359.09 | 298.57 | 5.93 |
|  |  |  | (217.85-315.69) | (292.02-436.95) | (227.67-369.46) | (5.7-6.16) |
| Ghana | 15 | 12,883 | 116.43 | 122.82 | 62.3 | 2.03 |
|  |  |  | (65.17-192.04) | (66.05-208.55) | (-3.94-128.53) | (1.48-2.58) |
| Other non-European | 121 | 106,137 | 114 | 171.96 | 111.43 | 2.84 |
|  |  |  | (94.6-136.22) | (138.03-211.71) | (74.94-147.93) | (2.6-3.08) |
| Total non-European | 681 | 316,720 | 215.02 | 273.22 | 212.69 | 4.51 |
|  |  |  | (199.17-231.79) | (252.21-295.51) | (190.13-235.26) | (4.37-4.65) |
| Total European | 116 | 169,814 | 68.31 | 82.36 | 21.83 | 1.36 |
|  |  |  | (56.45-81.93) | (67.86-99.03) | (5.06-38.6) | (1.14-1.58) |
| None (Ethnic-Dutch) | 292 | 386,521 | 75.55 | 60.53 | Ref. | Ref. |
|  |  |  | (67.13-84.73) | (53.65-68.04) |  |  |

^a^ Population on 1 April 2020

^b^ Standardised for age (in 15-year groups) and gender, using the total population of Amsterdam as the standard population

**Supplementary Table S5. Death rates by city district among those linked to the registration database, Amsterdam, The Netherlands, 15 June 2020-20 January 2021**

|  | **Deaths** | **Population ^a^** | **Crude rate per 100,00 population**  **(95% CI)** | **Standardised rate per 100,00 population ^b^**  **(95% CI)** | **Standardised rate difference (95% CI)** | **Standardised rate ratio**  **(95% CI)** |
| --- | --- | --- | --- | --- | --- | --- |
| **Total** | 291 | 873055 | 227,64 |  |  |  |
|  |  |  |  |  |  |  |
| **City district^c^** |  |  |  |  |  |  |
| Centre | 18 |  | 20,64 | 19,31 | Ref. | Ref. |
|  |  | 87223 | (12,23-32,61) | (11,39-30,63) |  |  |
| New-West | 75 |  | 46,84 | 44,8 | 25,49 | 2,32 |
|  |  | 160115 | (36,84-58,72) | (35,21-56,2) | (11,91-39,07) | (1,8-2,84) |
| North | 35 |  | 35,1 | 28,1 | 8,78 | 1,45 |
|  |  | 99705 | (24,45-48,82) | (19-52-39,16) | (-4,21-21,77) | (0,88-2,03) |
| East | 32 |  | 22,53 | 30,04 | 10,72 | 1,56 |
|  |  | 142011 | (15,41-31,81) | (20,5-42,47) | (-3,07-24,51) | (0,97-2,14) |
| West | 49 |  | 33,15 | 43,62 | 24,3 | 2,26 |
|  |  | 147831 | (24,52-43,82) | (32,18-57,79) | (9,06-39,55) | (1,71-2,8) |
| South | 51 |  | 34,82 | 27,35 | 8,04 | 1,42 |
|  |  | 146471 | (25,93-45,78) | (20,31-36,04) | (-3,71-19,79) | (0,87-1,96) |
| South-East | 31 |  | 34,56 | 36,07 | 17,76 | 1,87 |
|  |  | 89699 | (23,48-49,06) | (24,38-51,43) | (1,07-32,45) | (1,28-2,45) |

^a^ Population on 1 April 2020

^b^ Standardised for age (in 15-year groups) and gender, using the total population of Amsterdam as the standard population

^c^ 1 matched case had missing data

**Supplementary Table S6. Death rates by migration history (first and second generation combined) among those linked to the registration database, Amsterdam, The Netherlands, 15 June 2020-20 January 2021**

|  | **Deaths** | **Population ^a^** | **Crude rate per 100,00 population**  **(95% CI)** | **Standardised rate per 100,00 population ^b^**  **(95% CI)** | **Standardised rate difference (95% CI)** | **Standardised rate ratio**  **(95% CI)** |
| --- | --- | --- | --- | --- | --- | --- |
| **Total** | 292 | 873055 | 33,45 |  |  |  |
|  |  |  |  |  |  |  |
| **Migration background** |  |  |  |  |  |  |
| Netherlands Antilles | 5 | 12126 | 41,23 | 43,53 | 16,44 | 1,61 |
|  |  |  | (13,39-96,23) | (13,74-103,16) | (-22,69-55,58) | (0,7-2,51) |
| Morocco | 36 | 77213 | 46,62 | 70,81 | 43,73 | 2,61 |
|  |  |  | (32,66-64,55) | (48,39-100,04) | (18,76-68,69) | (2,23-3) |
| Surinam | 36 | 63944 | 56,3 | 55,89 | 28,8 | 2,06 |
|  |  |  | (39,43-77,94) | (38,9-77,77) | (9,76-47,85) | (1,69-2,43) |
| Turkey | 16 | 44417 | 35,02 | 66,14 | 39,06 | 2,44 |
|  |  |  | (20,59-58,5) | (37,26-108,58) | (5,62-72,49) | (1,92-2,97) |
| Ghana | 1 | 12883 | 7,76 | 8,85 | -18,24 | 0,33 |
|  |  |  | (0,2-43,25) | (0,22-49,31) | (-36,12-  -0,36) | (-1,62 - 2,29) |
| Other non-European | 13 | 106137 | 12,25 | 28,31 | 1,22 | 1,05 |
|  |  |  | (6,52-20,95) | (13,67-51,82) | (-16,72- 19,16) | (0,41-1,68) |
| Total non-European | 107 | 316720 | 33,78 | 53,66 | 26,57 | 1,98 |
|  |  |  | (27,69-40,82) | (43,68-65,24) | (15,21-37,93) | (1,73-2,23) |
| Total European | 32 | 169814 | 18,84 | 25,71 | -1,37 | 0,95 |
|  |  |  | (12,89-26,6) | (17,59-36,3) | (-11,28-8,54) | (0,57-1,33) |
| None (Ethnic-Dutch) | 152 | 386521 | 39,33 | 27,09 | Ref. | Ref. |
|  |  |  | (33,32-46,1) | (22,92-31,79) |  |  |

^a^ Population on 1 April 2020

^b^ Standardised for age (in 15-year groups) and gender, using the total population of Amsterdam as the standard population

**Supplementary Table S7. Hospitalisation rates under 60 years of age by migration history (first and second generation combined) among those linked to the registration database, Amsterdam, the Netherlands, 15 June 2020-20 January 2021**

|  | **Hospital admissions** | **Population ^a^** | **Crude rate per 100,00 population**  **(95% CI)** | **Standardised rate per 100,00 population ^b^**  **(95% CI)** | **Standardised rate difference (95% CI)** | **Standardised rate ratio**  **(95% CI)** |
| --- | --- | --- | --- | --- | --- | --- |
| **Total** | 419 | 715610 | 58,55 |  |  |  |
|  |  |  | (53,08- 64,44) |  |  |  |
| **Migration background** |  |  |  |  |  |  |
| Netherlands Antilles | 10 | 10044 | 99,56 | 101,8 | 78,69 | 4,41 |
|  |  |  | (99,56- 183,1) | (48,72- 187,46) | (15,22-142,16) | (3,74- 5,07) |
| Morocco | 90 | 68124 | 132,11 | 144,73 | 121,62 | 6,26 |
|  |  |  | (106,23- 162,39) | (116,22- 178,10 | (91,05-152,19) | (5,95- 6,58) |
| Surinam | 58 | 49825 | 116,41 | 101,79 | 78,68 | 4,41 |
|  |  |  | (88,39- 150,48) | (76,67- 132,5) | (51,22-106,14) | (4,05- 4,76) |
| Turkey | 66 | 39898 | 165,42 | 166,42 | 143,31 | 7,2 |
|  |  |  | (127,94- 210,46) | (128,66-211,78) | (102,74- 83,88) | (6,86- 7,54) |
| Ghana | 8 | 11056 | 72,36 | 90,72 | 67,61 | 3,93 |
|  |  |  | (31,24- 142,58) | (36,64- 186,36) | (0,47- 134,75) | (3,15- 4,7) |
| Other non-European | 69 | 96978 | 71,15 | 78,31 | 55,2 | 3,39 |
|  |  |  | (55,36- 90,05) | (60,65- 99,51) | (35,63- 74,78) | (3,05- 3,73) |
| Total non-European | 302 | 275925 | 109,45 | 112,22 | 89,12 | 4,86 |
|  |  |  | (97,45- 122,52) | (99,91- 125,63) | (75,32- 102,92) | (4,59- 5,12) |
| Total European | 46 | 146910 | 31,31 | 32,81 | 9,7 | 1,42 |
|  |  |  | (22,92-41,77) | (23,91-43,93) | (-1,36- 20,76) | (1,04- 1,8) |
| None (Ethnic-Dutch) | 69 | 292775 | 23,57 | 23,11 | Ref. | Ref. |
|  |  |  | (18,34-29,83) | (17,96- 29,27) |  |  |

^a^ Population on 1 April 2020

^b^ Standardised for age (in 15-year groups) and gender, using the total population of Amsterdam as the standard population
